# Sperm induce a secondary increase in ATP levels in mouse eggs that is independent of Ca^2+^ oscillations

**DOI:** 10.1042/BCJ20230065

**Published:** 2023-12-15

**Authors:** Cindy Ikie-Eshalomi, Elnur Aliyev, Sven Hoehn, Tomasz P. Jurkowski, Karl Swann

**Affiliations:** School of Biosciences, Cardiff University, Sir Martin Evans Building, Museum Avenue, Cardiff CF10 3AX, U.K.

**Keywords:** adenosine triphosphate, calcium signalling, sperm–egg interactions

## Abstract

Egg activation at fertilization in mouse eggs is caused by a series of cytosolic Ca^2+^ oscillations that are associated with an increase in ATP concentrations driven by increased mitochondrial activity. We have investigated the role of Ca^2+^ oscillations in these changes in ATP at fertilization by measuring the dynamics of ATP and Ca^2+^ in mouse eggs. An initial ATP increase started with the first Ca^2+^ transient at fertilization and then a secondary increase in ATP occurred ∼1 h later and this preceded a small and temporary increase in the frequency of Ca^2+^ oscillations. Other stimuli that caused Ca^2+^ oscillations such as PLCz1 or thimerosal, caused smaller or slower changes in ATP that failed to show the distinct secondary rise. Sperm-induced Ca^2+^ oscillations in the egg also triggered changes in the fluorescence of NADH which followed the pattern of Ca^2+^ spikes in a similar pattern to oscillations triggered by PLCz1 or thimerosal. When eggs were loaded with low concentrations of the Ca^2+^ chelator BAPTA, sperm triggered one small Ca^2+^ increase, but there were still extra phases of ATP increase that were similar to control fertilized eggs. Singular Ca^2+^ increases caused by thapsigargin were much less effective in elevating ATP levels. Together these data suggest that the secondary ATP increase at fertilization in mouse eggs is not caused by increases in cytosolic Ca^2+^. The fertilizing sperm may stimulate ATP production in eggs via both Ca^2+^ and by another mechanism that is independent of PLCz1 or Ca^2+^ oscillations.

## Introduction

At fertilization in mammalian eggs, the sperm activates development by causing a series of repetitive rises in the cytosolic Ca^2+^ concentration [1–3]. These Ca^2+^ oscillations represent one of the longest-lasting and well-characterized series of Ca^2+^ responses during cell stimulation. The Ca^2+^ oscillations during mammalian fertilization are initiated by sperm–egg membrane fusion which leads to the transfer of sperm-specific phospholipase C, PLCzeta (PLCz1), from sperm into the egg. PLCz1 triggers cycles of InsP_3_ production which lead to the repetitive Ca^2+^ increases or Ca^2+^ oscillations [2–5]. In mouse fertilization there is evidence for another late-acting factor that can cause a limited number of Ca^2+^ oscillations, but the mechanism of this effect is unclear [6,7]. The sperm-induced Ca^2+^ oscillations are of low frequency, with about one Ca^2+^ transient, or Ca^2+^ spike, about every 10–15 min. Measurements using dextran-linked Ca^2+^ dyes or the photoprotein aequorin suggest that the Ca^2+^ oscillations persist for up to 3 h [8,9]. The significance of Ca^2+^ oscillations has been demonstrated in mouse eggs where Ca^2+^ rises can be blocked by loading eggs with the Ca^2+^ chelator BAPTA. The inhibition of Ca^2+^ oscillations with BAPTA does not block sperm-egg fusion but it inhibits cell cycle resumption and exocytosis [1,10]. Stimuli that trigger Ca^2+^ oscillations can activate development up to the blastocyst stage, and hence Ca^2+^ oscillations are known to be necessary and sufficient for egg activation.

Cytosolic Ca^2+^ increases can stimulate mitochondrial activity in many somatic cell types. Ca^2+^ stimulates different mitochondrial matrix dehydrogenases and increases mitochondrial NADH and this leads to increases in ATP levels in HeLa cells, hepatocytes or pancreatic beta cells [11–15]. When the changes in ATP in these cell types are measured using firefly luciferase they involve a 20–30% increase in luminescence [11,14]. At fertilization in mouse eggs, the Ca^2+^ oscillations are also associated with Ca^2+^ increase in mitochondria and the stimulation of mitochondrial activity [16], as shown by transient changes in NADH and FAD fluorescence [16]. The Ca^2+^ oscillations at fertilization are also associated with an increase in ATP that was shown by monitoring the dynamics of ATP using firefly luciferase or using a genetically encoded fluorescent probes [9,17]. When measured with cytosolic firefly luciferase the increase in ATP involves about a 50–60% increase in luminescence, suggesting that it represents a large relative change in ATP. The ATP increase during fertilization is distinctive in that it generally involves two phases. The first increase in ATP occurs with the onset of Ca^2+^ oscillations, then after ∼1 h, there is a second phase of ATP increase [9,17]. Mouse eggs rely almost exclusively upon mitochondria for ATP production and luciferase targeting has shown that there is also a two-phased increase in mitochondrial ATP levels at fertilization. Complete inhibition of all Ca^2+^ oscillations with high concentrations of microinjected BAPTA blocks all increases in ATP at fertilization. However, the role of Ca^2+^ oscillations in causing the secondary ATP has not been investigated, and its mechanism remains unclear since there is no obvious extra stimulus for eggs at ∼1 h after sperm-egg fusion. It is not clear whether other stimuli that trigger Ca^2+^ oscillations in mouse eggs, such as thimerosal and Sr^2+^ [1,18], also cause the same two-step increase in ATP seen at fertilization.

In this study, we have investigated the role of Ca^2+^ oscillations in causing the two-phased increases in ATP at fertilization in mouse eggs. We show that the secondary increase in ATP is unique to fertilization induced Ca^2+^ oscillations and that it is not seen with PLCz1, or any other stimuli that increase Ca^2+^ in eggs. In addition, we find that the secondary rise in ATP still occurs when sperm-induced Ca^2+^ oscillations were blocked by low concentrations of BAPTA. Furthermore, the secondary increase in ATP is not explained by Ca^2+^ induced changes in NADH levels. We suggest that the fertilizing sperm may introduce a factor into the egg to stimulate ATP production in a Ca^2+^ independent manner.

## Results

### Ca^2+^ and ATP increases during fertilization

**Figure 1.**
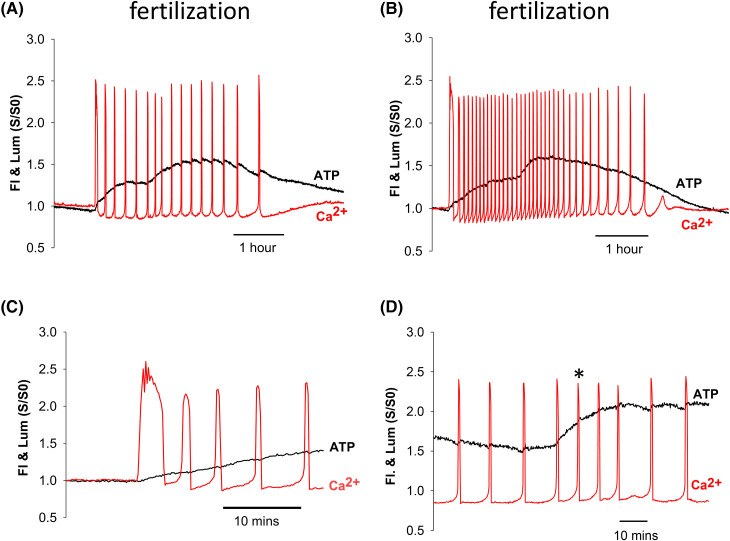
Sperm-induced increases in ATP at fertilization. Sample concurrent recordings of Ca^2+^ oscillations (fluorescence- ‘Fl’ — red trace) and ATP changes (luciferase luminescence- ‘Lum’ — black trace) in mouse eggs at fertilization are shown. The Fl and Lum traces are plotted as the ratio of the fluorescence or luminescence signals (S) to the signal before the start of changes (S0), as indicated by S/S0. (**A**) Shows a recording of Ca^2+^ oscillations at a typical frequency with a two-phased increase in ATP. (**B**) Shows a recording with a high frequency of Ca^2+^ oscillations, along with a two-phased change of ATP. The trace in (**C**) shows an expanded trace around the time of the first Ca^2+^ transients where the ATP increase starts at the same times as the Ca^2+^ rise (within 10s resolution). In (**D**) is shown an expanded scale of the timing of the second increase in ATP in another fertilizing egg. The trace starts with the sixth Ca^2+^ transient at fertilization. The start of the secondary rise in ATP is initiated before the first Ca^2+^ spike that signifies an increase in the frequency of Ca^2+^ oscillations (indicated by the asterisk*).

Our previous studies reported increases in ATP during Ca^2+^ oscillations at fertilization in mouse eggs using an image intensifier-based camera [9,17]. Figure 1 shows recordings of Ca^2+^ oscillations and ATP increases at fertilization using a CCD camera with a reduced noise in signal. Figure 1A is a typical recording where a series of 17 (±7.9 s.d., *n* = 37) Ca^2+^ oscillations, or repetitive Ca^2+^ spikes, were observed over a period of 183 min (±30 s.d. min), with a mean interval 14.0 min (±6 min 44 s s.d.), after adding sperm to the dish (Table 1). The initial Ca^2+^ increase at fertilization was consistently the largest as judged by the F/F0 ratio which was 2.3 (±0.20 s.d., *n* = 37). After the first Ca^2+^ increase there was a distinct shift in the inter-spike Oregon Green BAPTA dextran (OGBD) fluorescence to a level lower than that at the start of the recording, and then at ∼1 h there was a small and temporary increase in the frequency of Ca^2+^ oscillations. These eggs were found to contain two pronuclei at the end of the experiment and hence polyspermy was low under the conditions of our experiments.

**Table 1:** Changes in Ca^2+^ and ATP in eggs responding to different stimuli

Stimulus	Number of Ca^2+^ spikes & mean interval between spikes (Int ± the s.e.m.)	Amplitude of first/peak Ca^2+^ spike (F/F0)	Amplitude of ATP increase (% luminescence)
Fertilization *n* = 37	17 ± 7.9 Int = 840 ± 67s	2.3 ± 0.033	60 ± 3.4
PLCz1 *n* = 34	18 ± 7.7 Int = 958 ± 91s	2.7 ± 0.044	33* ± 2.9
Thimerosal *n* = 18	29 ± 15 Int = 200 ± 10s	2.1 ± 0.048	52 ± 6.7
Strontium *n* = 15	10 ± 6.3 Int = 1520 ± 260s	2.4 ± 0.073	31* ± 4.6
Fertilization + BAPTA *n* = 19	1	1.5 ± 0.052	68 ± 6.6
Thapsigargin *n* = 12	1	1.45 ± 0.031	11** ± 1.4
Thapsigargin + high Ca^2+^ *n* = 21	1	2.1 ± 0.043	5.7** ± 2128

The first column denotes the stimulus or conditions of stimulation and the number of eggs analyzed. The second column shows the number and intervals of Ca^2+^ transients. The third column shows the mean size of the largest Ca^2+^ increase estimated from the ratio of the peak OGBD fluorescence rise divided by the pre-stimulation fluorescence (F/F0). The last column shows the mean increases in ATP calculated from the peak % increase in luminescence signal. Variations of data ± are all standard errors of the mean − s.e.m. The asterixis indicate a statistically significant difference (*P* < 0.01) from control fertilization (*) or BAPTA fertilized eggs (**).

When Ca^2+^ oscillations were triggered by sperm there was a substantial increase in luciferase luminescence which (to within 10 s) was initiated as soon as the Ca^2+^ started to increase during the first Ca^2+^ spike (see Figure 1A–C). The increase in luminescence indicates an increase in ATP and it continued after the first Ca^2+^ spike. Then at 64 min ± 26 min s.d., *n* = 36/37) after the start of Ca^2+^ oscillations), there was a further phase of increased ATP. This second phase of ATP increase most often (in 31/36 eggs) started during a Ca^2+^ transient. Significantly this secondary ATP rise began before any increase in frequency of Ca^2+^ oscillations by ∼10 min (642 s, ±338 s s.d., *n* = 30), as illustrated by the trace in Figure 1D. After more than an hour, the luminescence signal peaked at 60% (±21% s.d., *n* = 37) above the pre-fertilization level (Table 1) and thereafter there was a decline towards the end of the series of Ca^2+^ oscillations. There were sometimes small increases in ATP that occurred during each Ca^2+^ spike, and these were generally more noticeable after the second phase of ATP increase. The secondary increase in ATP was evident in 36/37 fertilizing eggs, regardless of Ca^2+^ oscillation frequency, and by itself it involved a 23% (±9.2%, *n* = 36) increase in the luminescence of eggs over the initial luminescence values. Figure 1B shows recordings from a fertilizing egg that showed higher frequency Ca^2+^ oscillations respectively where there was also a secondary ATP increase ∼1 h into the oscillation series. These data confirm previous findings that a cytosolic ATP increase occurs in two distinct phases during fertilization of mouse eggs [9,17]. With better signal to noise, it is now clear the secondary phase of ATP increase occurs before any increase in the frequency of Ca^2+^ oscillations.

### Ca^2+^ and ATP changes in response to other stimuli

**Figure 2.**
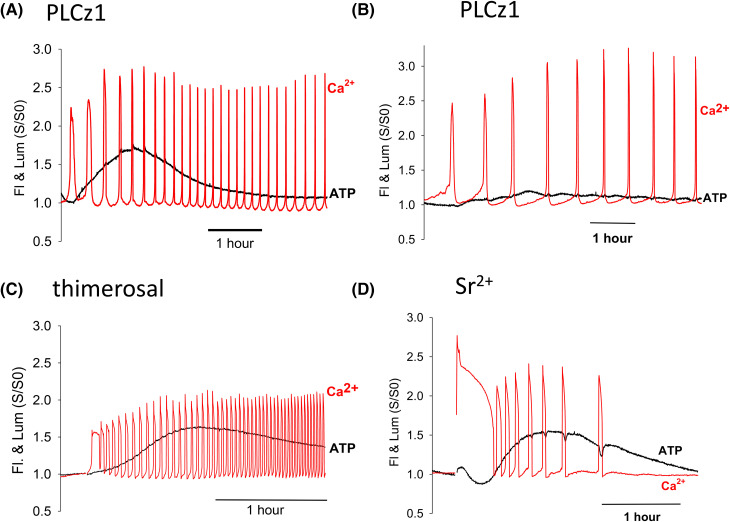
Ca^2+^ and ATP in response to different artificial stimuli. Recordings of Ca^2+^ (red) and ATP (black) changes in eggs stimulated by other stimuli that cause Ca^2+^ oscillations. As for Figure 1 the fluorescence and luminescence traces are plotted as the ratio of the signal (S) divided by starting signals (S0). Conditions are otherwise the same as in Figure 1. In (**A**) and example is shown of an egg with a relatively high frequency Ca^2+^ oscillations after injection of PLCz1 mRNA. In (**B**) is shown an egg also injected with PLCz1 mRNA that showed a lower frequency of Ca^2+^ oscillations. In (**C**) is shown an example of an egg treated with 10 µM thimerosal which was added just before the start of the trace. In (**D**) is an example of an egg that was treated with Sr^2+^ EGTA to induce Ca^2+^ oscillations.

**Figure 3.**
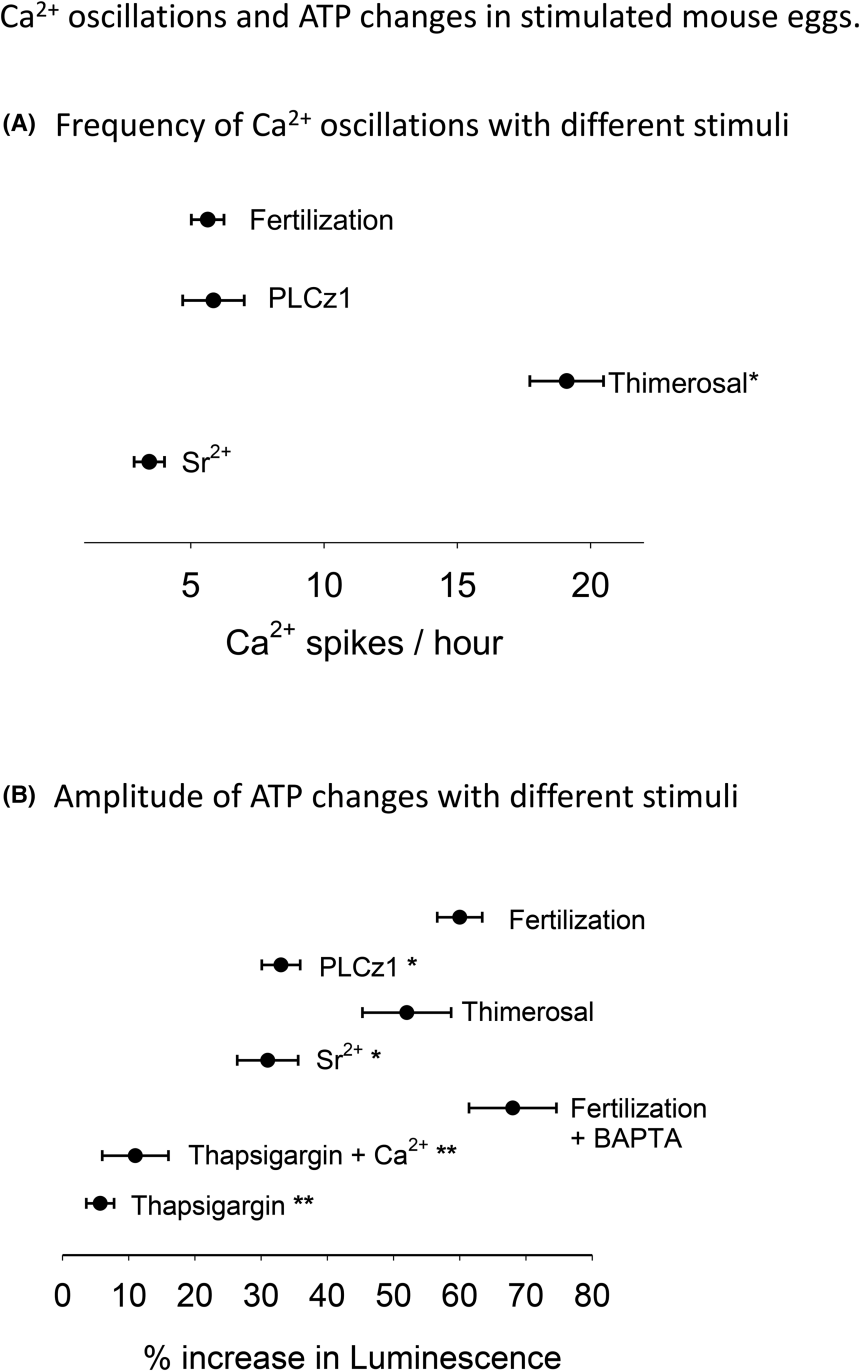
Ca^2+^ oscillations and ATP increases in stimulated mouse eggs. In (**A**) the mean frequency of Ca^2+^ oscillations, as Ca^2+^ spikes per hour, is plotted for fertilization, PLCz1 injection, and application of thimerosal and Sr^2+^ medium. Thimerosal responses show a significantly higher frequency of oscillations (*P* < 0.001) than fertilization. In (**B**) the amplitude of the Luminescence increases is plotted as the % if peak and compared with the luminescence before the start of Ca^2+^ signals. The mean change is shown with the standard errors of the mean. The asterixis indicate a statistically significant difference (*P* < 0.01) from control fertilization (*) or from fertilized BAPTA-treated eggs (**).

Previous studies show that Ca^2+^ plays a role in increasing mitochondrial activity at fertilization, but the secondary increase in ATP has no obvious cause. To establish how the fertilizing sperm might cause a secondary increase in ATP we investigated whether the pattern of ATP changes is reproduced by other stimuli that cause Ca^2+^ oscillations. Figure 2A and B show recording of Ca^2+^ oscillations after injection of PLCz1 RNA. These Ca^2+^ oscillations started soon after placing the eggs on the imaging system and persisted for >4 h, with a similar frequency of Ca^2+^ oscillations to those seen at fertilization (mean interval 14 min 27 s, ±9 min 22 s, *n* = 34 eggs, see Figure 3A). During the Ca^2+^ oscillations the luminescence of luciferase increase started during the first Ca^2+^ spike indicating an increase in ATP. The ATP then increased gradually over ∼2 h reaching a peak at 33% (±17 s.d., *n* = 34) above the starting luminescence which is about half the size of the increase at fertilization (see Figure 3 and Table 1). There were small increases in luminescence during each Ca^2+^ spike but we did not observe any secondary increases in ATP in any egg regardless of the frequency of Ca^2+^ oscillations. The second change in ATP was not due to activation related events because all these PLCz1 injected eggs were activated and showed distinctive second polar bodies similar to fertilized eggs. There was also no clear sign of a temporary increase in the frequency of Ca^2+^ oscillations at ∼1 h. These data suggest that PLCz1 does not mimic the sperm with respect to the secondary increase in ATP.

We also examined the effect of Ca^2+^ oscillations induced by thimerosal that triggers high frequency Ca^2+^ oscillations in mouse eggs [18]. Low concentrations of thimerosal addition to eggs caused high frequency Ca^2+^ oscillations (mean interval 3 min 13 s, ±1 min 5 s, *n* = 18) and a gradual rise in luciferase luminescence (Figure 2C). There was considerable variation in the amplitude of the ATP rise but the increase was always slower and smaller at 52% (±28%, *n* = 18) than the change in fertilizing mouse eggs (see Figure 3 and Table 1). The increases in Ca^2+^ sometimes (in 7/19 eggs) lead to a small short lasting decreases in ATP, but there was no sign of a secondary rise in ATP.

We also added Sr^2+^ to eggs and measured Ca^2+^ and ATP changes. Addition of Sr^2+^ containing medium to eggs also lead to a series of Ca^2+^ oscillations, but these started with a prolonged Ca^2+^ transient followed by lower frequency oscillations (Figure 2D) (mean interval 20 min 40 s, ±11 min 20 sec, *n* = 15). The initial large Ca^2+^ rise (F/F0 ratio of 2.4 ± 0.27, *n* = 15) was associated with a decrease in luciferase luminescence in all eggs followed by a recovery in luminescence levels before a slower decline. As with thimerosal, individual Ca^2+^ transients were associated with small declines in luminescence. These small and short duration decreases in ATP occurred in most recordings with Sr^2+^ (12/15 eggs). Such decreases in ATP were also associated with a rapid ‘rebound’ after each Ca^2+^ transient was terminated (see Supplementary Figure S1). There was no sign of a two-phased increase during the rising phase of luminescence and the overall ATP increase was significantly smaller than that seen at fertilization at 31% (±17%, *n* = 15) (see Figure 3 and Table 1). These data show that an increases in ATP can be stimulated by Ca^2+^ oscillations. However, they also suggest that the distinct two phases of ATP increase are not mimicked by other stimuli and that, even with higher frequency Ca^2+^ oscillations, other stimuli fail to match the size of the overall increase in ATP seen at fertilization.

### ATP changes in fertilized eggs loaded with BAPTA

**Figure 4.**
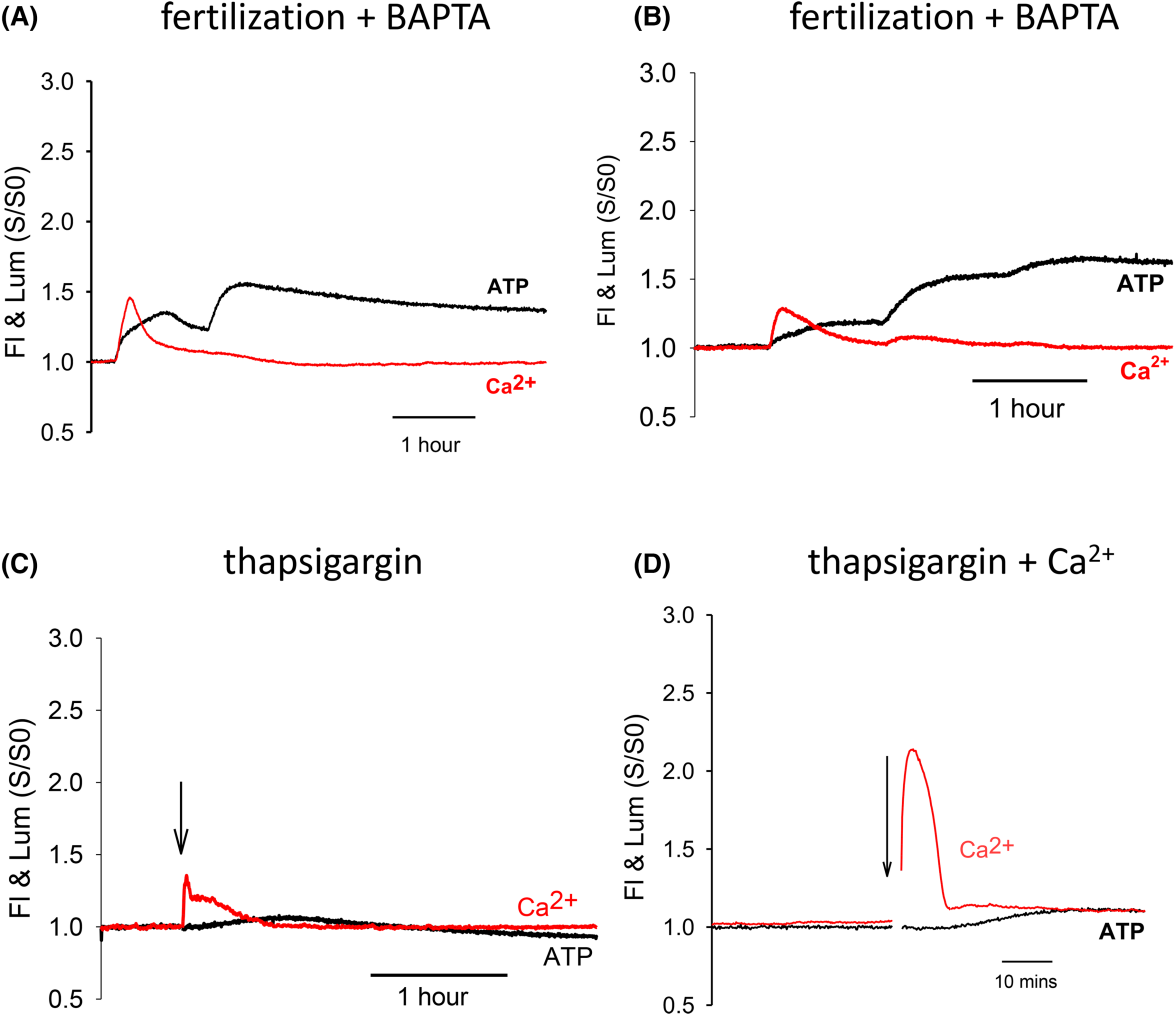
Ca^2+^ and ATP changes in BAPTA-treated eggs and in eggs treated with thapsigargin. Recordings of Ca^2+^ and ATP in (**A**) and (**B**) are shown for two different eggs that were fertilized after they had been preloaded with BAPTA. Conditions and plots are otherwise the same as Figure 1 and 2. In (**A**) there was a single and small Ca^2+^ transient and this was followed by a two-phased increase in ATP levels. (**B**) shows another egg where there was a similar small initial Ca^2+^ transient and then several phases of increases of ATP levels. In (**C**) is shown a recording in which 10 µM thapsigargin was added to an egg (at the arrow) which causes a single small increase in Ca^2+^ which was associated with a small and delayed increase and then a decrease in ATP levels. In (**D**) is shown a recording from an egg where the addition of addition of thapsigargin was combined with additional CaCl_2_ (at the arrow) to increase the extracellular Ca^2+^ up to 5 mM. This led to a single and larger increase in Ca^2+^ levels and then a small and delayed rise in ATP.

The Ca^2+^ chelator BAPTA has been previously used to inhibit Ca^2+^ oscillations in eggs. We used low concentrations of BAPTA-AM to block Ca^2+^ oscillations which allows for the occurrence of an initial Ca^2+^ transient which provides a marker of sperm-egg interaction and fusion [1,10]. Figure 4A shows a typical recording of Ca^2+^ and ATP during fertilization of low BAPTA loaded eggs. There is a single Ca^2+^ transient that was significantly smaller than the initial Ca^2+^ transient during control fertilization with an F/F0 of 1.5 ± 0.23 (*n* = 19) (Figure 3 and Table 1). At the same time as the rise in Ca^2+^ the luciferase luminescence showed a substantial increase in amplitude indicating an increase in ATP. Then after a time interval of 59 min (±15 min, *n* = 19) after the initial Ca^2+^ transient, there was marked secondary increase in ATP that increased the signal to 68% (±29%, *n* = 19) above pre-fertilization levels. In some eggs (7/19) there were additional increases in ATP (Figure 4B). Whenever we observed these increases secondary in ATP there was no distinct Ca^2+^ increase. These data show that the size of secondary rise in ATP is not dependent upon any changes in Ca^2+^ beyond the first Ca^2+^ increase. In addition, the timing of the sperm-induced second change in ATP is remarkably similar between control and BAPTA loaded eggs. This suggests that the timing and amplitude of the second phase of ATP increase is independent of ongoing Ca^2+^ oscillations.

The single Ca^2+^ increase that we observed during fertilization of BAPTA loaded eggs could be mimicked by other stimuli that have been reported caused a single Ca^2+^ increase. Figure 4C shows Ca^2+^ and ATP levels in eggs treated with 10 µM thapsigargin. Some eggs showed a very small increase in Ca^2+^ with thapsigargin (<1.3 F/F0). However, if we only consider eggs with a larger increase in Ca^2+^, where the average increase is similar to fertilized BAPTA loaded eggs, the single thapsigargin-induced Ca^2+^ rise only caused a very small and delayed increase in ATP levels in eggs (11% ±5.0% *n* = 12). This was followed by a gradual decline in ATP. We could increase the amplitude of the initial Ca^2+^ increase (F/F0 = 2.1% ±0.20%, *n* = 21) by raising the extracellular Ca^2+^ at the same time as adding thapsigargin. The larger Ca^2+^ increase still only caused a very small and slow increase in ATP levels (to 5% ±9.5%, *n* = 21) that was significantly smaller than the ones seen in fertilizing BAPTA loaded eggs (Figure 3 and Table 1). Most of these eggs in high Ca^2+^ lysed after a couple of hours but the ATP increase had already peaked, and it never increased beyond a 10% increase in luminescence. These data suggest that a single Ca^2+^ transient seen during fertilization of BAPTA loaded eggs cannot account for the initial ATP stimulated by the sperm and that the secondary increase in ATP levels is not caused by, or dependent upon, any increase in cytosol Ca^2+^ levels.

### Redox state changes in eggs during Ca^2+^ oscillations

**Figure 5.**
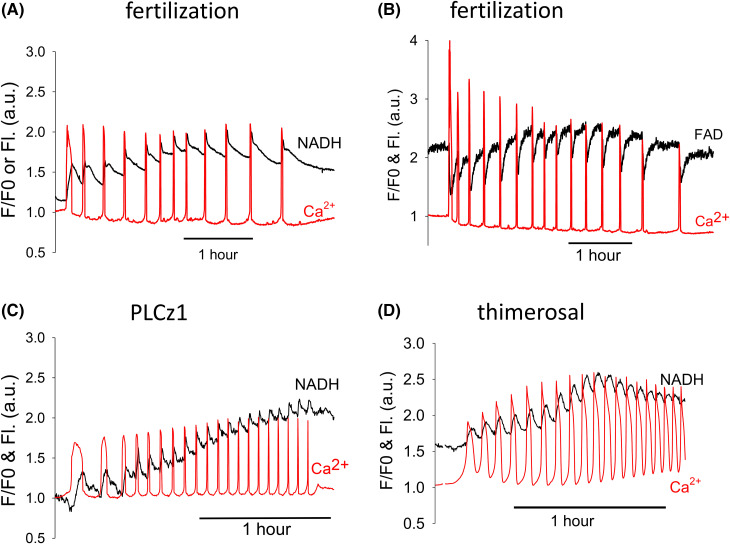
Changes in autofluorescence in eggs in response to Ca^2+^ oscillations initiated by different stimuli. Recordings are shown of Ca^2+^ alongside changes in NADH or FAD fluorescence in eggs stimulated to undergo Ca^2+^ oscillations by sperm, PLCz1 or thimerosal. Ca^2+^ traces are plotted as S/S0 as in Figure 1, whereas the autofluorescence from NADH or FAD are plotted in arbitrary units (a.u.). (**A**) and (**B**) show examples of Ca^2+^ oscillations at fertilization with concurrent recording of NADH (from a set of *n* = 22 eggs) or for FAD (from a set of *n* = 18 eggs) were there was an increase in frequency after ∼1 h into the response. Each Ca^2+^ transient is accompanied by a corresponding increase in NADH fluorescence (**A**) or decreases in FAD fluorescence (**B**). In (**C**) and (**D**) are shown examples of Ca^2+^ oscillations and increases in NADH in eggs injected with PLCz1 mRNA (as in Figure 2) or treated with 10 µM thimerosal. Each increase in Ca^2+^ is again associated with increase in NADH levels. These are samples from 11 eggs for PLCz1 and 20 eggs for thimerosal.

It has been shown that there are Ca^2+^ dependent changes in mitochondrial redox state of NADH and FAD during fertilization in mouse eggs and these could underlie the changes in ATP [16]. Previous recordings of NADH and FAD autofluorescence at fertilization did not extend beyond 1 h, so we measured these redox changes alongside Ca^2+^ oscillations for over 4 h. We could not record autofluorescence at the same time as luciferase luminescence because luciferin is fluorescent. However, we could monitor autofluorescence alongside Ca^2+^ oscillations. Figure 5A and shows changes in NADH fluorescence during Ca^2+^ induced by fertilization. Figure 5B shows similar change in FAD fluorescence. There were distinct increases in NADH fluorescence, or decreases in FAD, associated with each Ca^2+^ spikes at fertilization. With NADH the increases fluorescence did not recover to baseline values before the next Ca^2+^ which meant there was some ‘integration’ of the NADH fluorescence during the Ca^2+^ oscillations. When the frequency of Ca^2+^ oscillations increased, the peaks of NADH increased but this followed, rather than preceding, the increase in Ca^2+^ spike frequency. The secondary ATP increase is expected to occur just ahead of the increase in frequency of Ca^2+^ oscillations (see Figure 1C). When we measured the amplitude of each of the NADH increases at these times we could not find any evidence for a change in the amplitude of the NADH rises that occurred at the time of the temporary frequency increase (see Supplementary Figure S2). We also found that NADH fluorescence change in fertilized eggs were very similar to those seen in PLCz1 injected eggs, or in eggs treated with thimerosal (Figure 5C,D). With FAD there was no sign of integration of the transients decreases in signal. Hence, unlike changes in ATP, the changes in NADH or FAD in fertilizing eggs appear to be caused by individual Ca^2+^ transients and there were no additional changes ∼1 h into the recordings when the secondary ATP increase typically occurred.

## Discussion

We have previously showed that there is a distinct increase in ATP levels, as indicated by luciferase luminescence, in fertilizing mouse eggs [9,17]. Our current study used a camera with less ‘noise in signal’ that allowed for a better resolution of Ca^2+^ and ATP signals. It is clear that the ATP increase starts at the same time as the first Ca^2+^ increase. This rapid change in ATP is not seen with other agents that cause Ca^2+^ release in eggs. In addition, we confirmed that the ATP increase in fertilizing mouse eggs generally occurs in two phases, with a secondary ATP increase occurring ∼1 h after the start of the initial increase. This secondary increase in ATP occurs ∼10 min before any change in the frequency of Ca^2+^ oscillations, and that it occurs independently of any ongoing Ca^2+^ oscillations. Thimerosal causes high frequency Ca^2+^ oscillations in eggs and was the most effective artificial stimulus for elevating ATP, but thimerosal failed to cause any secondary increase in ATP. Most notably the secondary changes in ATP seen at fertilization could not be mimicked by PLCz1 which is considered the physiological stimulus of the initial series of Ca^2+^ oscillations at fertilization. Hence, our findings imply that sperm-induced Ca^2+^ oscillations involve another mechanism for inducing the secondary rise in ATP. It is not clear why the sperm causes increased in ATP, and this is difficult to address directly because agents that inhibit mitochondrial ATP production rapidly leads to sustained Ca^2+^ increases [16,17]. However, our current data suggest that the secondary ATP increase could promote Ca^2+^ release at fertilization because it precedes a small and transient increase in the frequency of Ca^2+^ oscillations and the concentration of ATP can affect Ca^2+^ release in mouse eggs [19]. ATP can also be seen as an indicator of bioenergetic changes at fertilization, and our data clearly suggests that the sperm stimulates ATP generating mechanism in eggs that are not entirely due to cytosolic Ca^2+^ increases.

We previously found that ATP changes were dependent upon Ca^2+^ by injecting sufficient of BAPTA to suppress all Ca^2+^ changes at fertilization. However, this may have reduced the level of Ca^2+^ in the egg such that mitochondria activity is generally suppressed, or else could have affected sperm–egg incorporation. Here, we have used low concentrations of BAPTA to eliminate all but the first Ca^2+^ transient at fertilization, which gives an unequivocal marker of the timing of sperm-egg fusion [10]. It was notable that these BAPTA loaded eggs still showed both the initial and the secondary rise in ATP, and in some cases further increases in ATP. The initial ATP increase occur at the same time as a Ca^2+^ rise, but it was consistently larger than seen with other stimuli that caused a single Ca^2+^ rise. Hence, it is unclear how much of this initial ATP increase can be explained by a Ca^2+^ rise. More significantly, in BAPTA loaded eggs, when there was a distinct secondary rise in ATP it occurred the same time, and of similar amplitude, to control fertilization eggs in the absence of a Ca^2+^ rise. This suggest that these secondary ATP increases occur in the same way as normal fertilization despite the lack of ongoing Ca^2+^ oscillations. It is possible that further increase in ATP were due to extra sperm fusing with the eggs and represent an initial sperm-induced Ca^2+^ stimulus. We cannot monitor the timing of sperm-egg fusion at the same time as measuring Ca^2+^ and ATP. Nevertheless, any extra sperm-egg fusion event would have caused an ATP increase without a Ca^2+^ rise, hence the responses still cannot be explained by Ca^2+^ rise. Hence, we can still conclude that sperm–egg fusion stimulates a substantial part of the ATP increase in mouse eggs in a way that is independent of Ca^2+^ increases.

It was notable that the second increase in ATP occurs ∼1 h after the initial Ca^2+^ rise in both normally fertilizing eggs and BAPTA loaded eggs. This timing is around the time of second polar body emission in fertilizing eggs, but second polar body emission occurred in all PLCz1 injected eggs, where there was no secondary increase in ATP. Hence, the secondary increase in ATP is not related to events of meiotic resumption. A rise in ATP in itself could be due to an increase in ATP production or a decrease in ATP usage. There is evidence that a sudden reduction in ATP usage can lead to an increase in ATP from our data. For the later Sr^2+^ induced Ca^2+^ oscillations there was a small ATP decrease, and then an increase in ATP, each time the later Ca^2+^ transients decline (see Figure 2D and Supplementary Figure S1). This may have happened with Sr^2+^ because it is an unphysiological stimulus for the IP3R. The pumping of Ca^2+^ into the endoplasmic reticulum is likely to consume a significant amount of ATP, and the sudden decline in this demand may have led to a rebound increase in ATP. However, such short term ‘rebound’ increases lasted for ∼1 min. It is unclear how this type of readjustment could lead to the hour long increases in ATP seen with the secondary rise.

It was also reported previously that increases in NADH and FAD occur with each Ca^2+^ transient in fertilizing mouse eggs. This is similar to Ca^2+^ induced NADH changes reported in somatic cell such as hepatocytes [20]. The previous recordings of NADH and FAD were too short to indicate whether there is any secondary increase in mitochondria redox state at fertilization, so we recorded Ca^2+^ oscillations for over 3 h. The NADH increased with each Ca^2+^ spike, and there was a gradual rise in resting NADH levels after 1 h. We quantified the NADH changes around this time and it was clear that NADH peaks did not increase in amplitude ahead of the increase in frequency of Ca^2+^ oscillations. Hence for NADH there was no evidence for increased mitochondrial redox changes that might account for the secondary ATP increase. We found a similar result for changes in FAD in fertilizing eggs, where each FAD decrease was simply tracking each Ca^2+^ transient throughout the fertilization response. The source of NADH in mitochondria is from several mitochondrial matrix dehydrogenases that are known to be Ca^2+^ dependent. The FAD in mitochondria mostly arise from parts of the pyruvate dehydrogenase complex, and pyruvate dehydrogenase activity is, indirectly, stimulated by Ca^2+^. Hence, the lack of any secondary alteration in NADH or FAD at ∼1 h is consistent with the idea that the secondary increase in ATP is not Ca^2+^ dependent. Our previous studies showed that the first and secondary ATP increase occur within the mitochondrial matrix. Since it does not appear to involve additional NADH, it is possible that the secondary ATP increase is caused by stimulation of enzyme complexes that are downstream of Complex I such as cytochrome *c* oxidase or the ATP synthase which can be rate-limiting steps in oxidative phosphorylation [21]. Our current data, along with previous studies, suggest that fertilization of mouse eggs involves both a Ca^2+^ dependent and a Ca^2+^ independent stimulation of ATP production. The sperm itself is highly metabolically active compared with the egg. Sperm-derived enzymes involved in glycolysis as well as the sperm mitochondria will enter the egg after gamete fusion, and these could contribute to ATP production. However, the sperm volume and mitochondrial mass are >1000 times smaller than those of the egg so it seems unlikely that sperm mitochondria themselves, or sperm glycolytic enzymes stimulate the ATP increase. There is evidence that the fertilizing sperm stimulates glycolysis during mouse fertilization in a Ca^2+^ independent manner [22], but mitochondria appear to provide the vast majority of the ATP for fertilizing eggs. Hence, it seems more likely that the ATP increase in mouse fertilization is partly due to a sperm-derived factor egg stimulating egg mitochondria. It has previously been suggested that sperm contain a late-acting factor, in addition to PLCz1, that can cause a limited Ca^2+^ release in eggs at ∼1 h after fusion [7]. Future studies should determine what the relationship might be between these proposed factors delivered by the fertilizing sperm to stimulate changes associated with egg activation.

## Materials and methods

### Gamete collection and microinjection

Female CD1 mice, or 6–8 weeks age, were super-ovulated by two serial intraperitoneal injections of PG600 ∼48 h apart. Mice were maintained in environmentally enriched cages. All procedures on mice were reviewed and approved by the Animal Welfare and Review Body at Cardiff University and carried out in the Biological Services Unit in the School of Biosciences at Cardiff University. Procedures were in compliance with, and under the authority, of a UK Home Office Licence held by K.S. at Cardiff University. Mouse eggs were collected from female mice that were culled by cervical dislocation 15 h after the second hormone injection [5,9,16]. Eggs were release from the oviducts and separated from cumulus cells by ∼5 min incubation with hyaluronidase in M2 medium (Sigma-Merck, U.K.) as described previously [5,9]. Male mice, of 4–6 months age, were culled by cervical dislocation and sperm collected from the cauda epididymis. Sperm were released into a T6 based medium and capacitated for 2 to 3 h in an incubator with 5% CO_2_ at 37°C before addition to the drop of medium containing the eggs.

Mouse eggs were microinjected with solutions containing both recombinant firefly luciferase, to measure ATP, and OGBD, to measure cytosolic Ca^2+^. In cases where autofluorescence was measured, either OGBD (for NADH) or else Rhod2 dextran (for FAD) was used to measure cytosolic Ca^2+^. Recombinant firefly luciferase (Sigma–Aldrich, U.K.) was made up at a stock concentration of 10 mg/ml in KCl HEPES (120 mM KCl, 20 mM HEPES, pH 7.2), and OGBD (Thermo-Fisher, U.K.) at a stock of 1 mM, also in KCl HEPES. These solutions were microinjected together in a ratio of 2 : 1 (luciferase: OGBD). The microinjection used an electrically assisted method that we have described in detail previously [23]. It consists of inserting a micropipette using voltage oscillations on an electrical amplifier attached to inside of the micropipette and the drop containing the eggs. Pressure pulses are applied to the back micropipette holder to inject solutions (∼10 pl) into the eggs. Following microinjection, the zona pellucidae were removed from eggs by brief exposure to acid Tyrode's solution (Sigma–Aldrich, U.K.). The eggs were then pipetted into drops of HKSOM medium in a heated microscope stage device at 37°C. The eggs adhered to the glass coverslip that formed the base of the drop that was then used for imaging.

### Medium and reagents

Eggs were maintained in M2 medium (Sigma–Aldrich, U.K.) at 37°C under drops of mineral oil in plastic petri dishes. For all imaging studies eggs were maintained in a HEPES buffered KSOM medium (HKSOM) as described previously [9,16]. The HKSOM did not contain any bovine serum albumin which allows the zona-free eggs to adhere to the glass covers slips that formed the base of the imaging chamber. For fertilization experiments, the glucose concentration in HKSOM was increased to 5 mM. For all recordings of luciferase luminescence, the eggs were incubated in a medium containing 100 µM sodium luciferin (Sigma–Aldrich, U.K.). The luciferase injected eggs were allowed to equilibrate with luciferin medium for ∼60 min, until light emission stabilized, before the start of recordings. Spermatozoa were capacitated in a T6 like medium with supplements [24]. Microinjection solutions were made up of a KCl-HEPES buffer of 120 mM KCl, 20 mM Na-HEPES, at pH 7.2. Stocks of chemicals added to eggs were made up at 1000 times the working concentration and added to the dish containing eggs at a 10× concentration. Sr^2+^ medium consisted of a mixture of 100 mM SrCl_2_ and 20 mM EGTA. This was also added (with 1 in 10 dilution) to the HKSOM medium containing the eggs which then leads to the EGTA chelating the Ca^2+^ in the HKSOM medium, leaving a free Sr^2+^ of 8 mM. Human PLCz1 template DNA was synthesized by Twist Biosciences and inserted into pET28a vector within NdeI/XhoI restriction sites. PLCz1 mRNA used for injections, was synthesized from linearized plasmid DNA using the mMessage Machine T7 kit (Ambion) and a poly(A) tailing kit (Ambion), following the manufacturer's instructions. The PLCz1 mRNA (∼0.01 mg/ml) was mixed with OGBD and luciferase and injected with the same micropipette.

### Live cell imaging

Mouse eggs were imaged using inverted epifluorescence microscopes. The system for measuring OGBD fluorescence and luciferase luminescence consisted of a Zeiss Axiovert 100 microscope equipped with a 20× 0.75 NA Fluar objective, with fluorescence excitation light provided by a white LED and control box (MonoLED, Cairn Research Ltd, U.K.). Excitation light passed through a 450–490 nm bandpass filter and was reflected onto the eggs with a 505 nm dichroic mirror. Both the fluorescence and luminescence light passed through a 510 nm long-pass filter before being projected onto the camera which was a Retiga LUMO^TM^ CCD camera (Teledyne Photometrics, U.S.A.), with pixel binning of 16 × 16. Micromanager software (Version 2.0 https://micro-manager.org/) was used to record images and control the fluorescence excitation. To monitor Ca^2+^ and ATP levels in eggs we continuously switched between fluorescence and luminescence imaging modes. With the Retiga LUMO^TM^ camera, luminescence was collected for 9.9 s and fluorescence signal collected during a 100 ms pulse of light from the LED. The signals were separable because the fluorescence was 100 to 1000 times greater than the luminescence. The luciferase signal is initially high after placing eggs in luciferin due to the ‘flash kinetics’ of luciferase, so in all experiments the eggs were incubated in luciferin for >1 h in order for luciferase signals to stabilize before any additions. Traces were excluded if the luminescence signal was unstable before the start of Ca^2+^ change. The % changes in luminescence signals were taken from the value in the minute before the start of any Ca^2+^ response. The entire imaging system was housed in a light-tight dark box during imaging runs. The door was opened briefly during the experiments to add sperm or media and the artefacts in recordings that this caused were not plotted.

Autofluorescence from eggs was measured by excitation with UV light or blue light for NADH and FAD, respectively. The blue autofluorescence signal is labelled as ‘NADH’ since this NAD(H) pool is >50 times greater than that for NADP(H) in mouse eggs [25]. For experiments that measured NADH or FAD fluorescence in eggs, a Nikon Eclipse microscope was used. Images were collected with a 20× 0.75 NA Plan Apo objective with excitation light provided by white or UV LED lamps (MonLED, Cairn Research Ltd). Images were collected with exposure durations of 100 ms with 8 × 8 pixel binning. NADH was excited using a UG11 excitation filter and collected with a 450–490 bandpass emission filter. FAD fluorescence used a 440 nm (10 nm width) bandpass filter for excitation and a 520–550 nm emission filter. Further details on the fluorescence imaging system for eggs are described elsewhere [5]. The only change was to use a Retiga R3^TM^ camera (Teledyne Photometrics, U.S.A.) to record fluorescence images and use Micromanager software for data acquisition.

### Data analysis

All fluorescence image files consisted of stacks of tif files that were analyzed using ImageJ (https://imagej.nih.gov/ij/download.html). The fluorescence and luminescence intensities of eggs were extracted using the Multi-measure plugin. SigmaPlot12.0 was used to subtract background fluorescence or luminescence signals before plotting and analyzing recordings. The OGBD fluorescence and the luciferase luminescence traces are plotted as S/S0 which is the signal intensities over time divided by the signal of each egg before the addition drug or start of fertilization. Summary data from traces are reported in the main text as means and standard deviations (s.d.), and in Figure 3 and in Table 1 as means and standard errors of the mean (s.e.m.) with ‘n’ numbers referring to eggs, with at least two and three or four experimental runs on different days. Statistical significance was estimated using a Student's *t*-test, or else a Mann–Whitney Rank sum test if the data was not normally distributed.

## Data Availability

The datasets underlying this article will be shared upon a reasonable request to the corresponding author.

## Open Access

Open access for this article was enabled by the participation of Cardiff University in an all-inclusive *Read & Publish* agreement with Portland Press and the Biochemical Society under a transformative agreement with JISC.

## Abbreviations

ATP: adenosine triphosphate
BAPTA: 1,2-bis-(2-aminophenoxy)ethane-N,N,N′,N′-tetra acetic acid tetra(acetoxymethyl) ester
FAD: flavin adenine dinucleotide
NADH: reduced nicotinamide adenine dinucleotide
OGBD: Oregon Green BAPTA dextran
PLCz1: phospholipase Czeta1
