# Supplementary material for: Sperm induce a secondary increase in ATP levels in mouse eggs that is independent of Ca2+ oscillations
Source: Biochem J. 2023 Dec 15;480(24):2023–35. doi: 10.1042/BCJ20230065 (PMC10754276; doi:10.1042/BCJ20230065)
Supplement: Supplementary Material [file BCJ-480-2023-s1.pdf]

Sr^{2+} induced Ca^{2+} oscillations

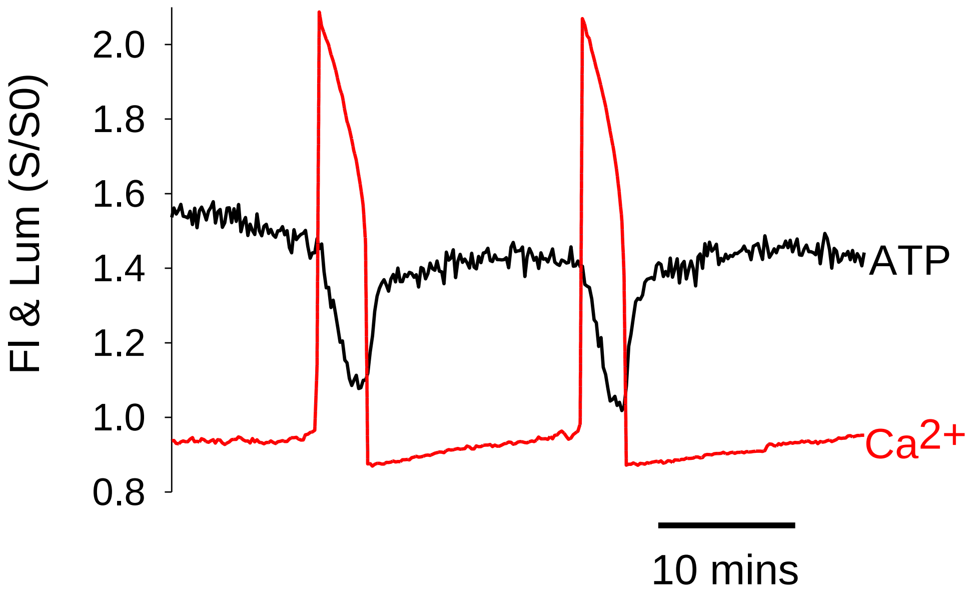

Supplementary Fig.1. An example of Ca^{2+} and ATP measurements in a mouse egg responding to medium containing Sr^{2+}. The conditions for recordings are the same as those described in Fig.2D but this is from a different egg with the trace starting 2 hours and 50 minutes after the addition of Sr^{2+}. The trace has an expanded timescale which shows a phenomenon where the ATP decreased during Ca^{2+} rises, and then rebounded back up as soon as the Ca^{2+} transient was terminated. We observed this effect in 12/15 of the Sr^{2+} treated eggs as well as in 7/19 of the thimerosal treated eggs. We did not see it in eggs that were undergoing Ca^{2+} oscillations at fertilization or after PLC1z injection.

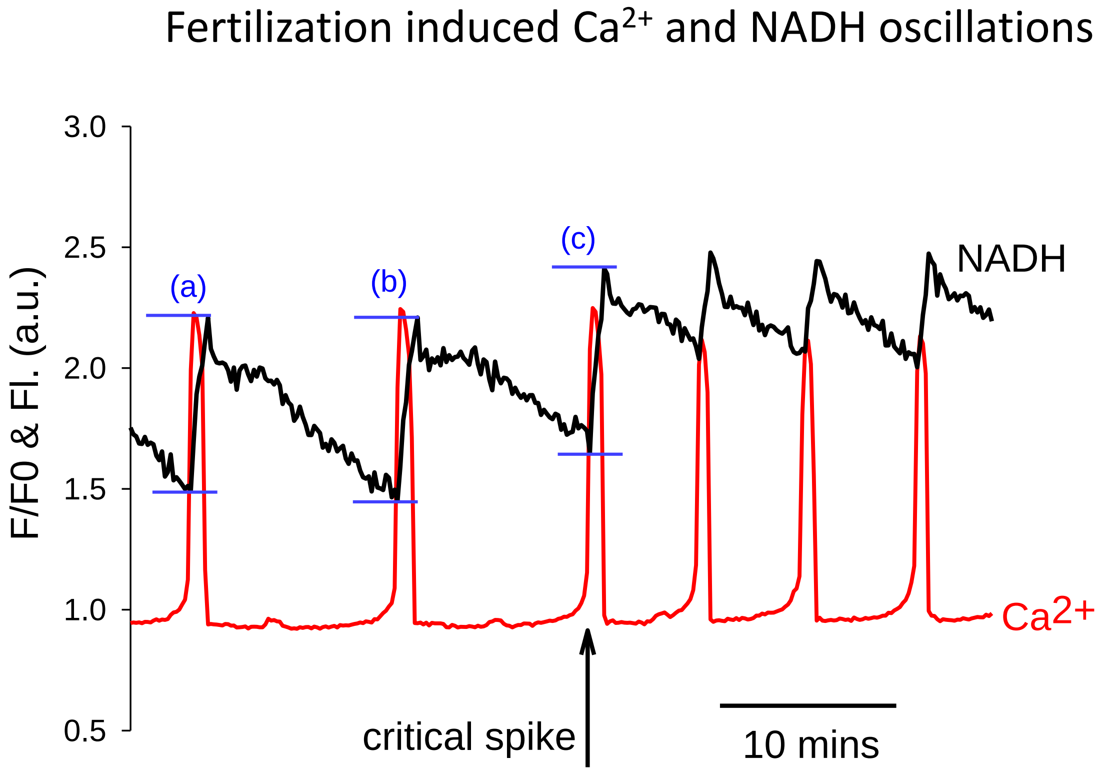

Supplementary Fig.2. We quantified the changes in NADH in the time period leading up to the increase in frequency of Ca^{2+} oscillations at around 1 hour into fertilization. The speed up of Ca^{2+} oscillations can be used as a marker for when the secondary ATP increase starts because in our luminescence recordings the secondary ATP increase started during the first Ca^{2+} spike in the series (see Fig.1C). This is labelled as the 'critical spike' in the above Figure. We measured the magnitude of each NADH increase by the differences between the blue bars in (a), (b) and (c). When were compared the amplitudes of the changes in NADH across n=19 eggs we found that the change in amplitude was +2.7% (sem=3.7%) from spike (b) compared to (a), and the amplitude was -0.28% (sem=3.8%) from spike (c) compared to (b). Using a paired t test for the data has a p=0.61. We could not find evidence for a difference in the amplitude of NADH increases leading up to the increase in Ca^{2+} oscillation frequency.
